# Chemical Ecology of the Colorado Potato Beetle, *Leptinotarsa decemlineata* (Say) (Coleoptera: Chrysomelidae), and Potential for Alternative Control Methods

**DOI:** 10.3390/insects4010031

**Published:** 2012-12-20

**Authors:** Ludovic Sablon, Joseph C. Dickens, Éric Haubruge, François J. Verheggen

**Affiliations:** 1 Unité d’Entomologie fonctionnelle et évolutive, Gembloux Agro-Bio Tech, Université de Liège, Passage des Déportés 2, B-5030 Gembloux, Belgium; E-Mails: e.haubruge@ulg.ac.be (E.H.); fverheggen@ulg.ac.be (F.J.V.); 2 Invasive Insect Biocontrol and Behavior Laboratory, Plant Sciences Institute, Henry A. Wallace Beltsville Agricultural Research Center, Agricultural Research Service, United States Department of Agriculture, Beltsville, Maryland, United States of America 20705; E-Mail: joseph.dickens@ars.usda.gov

**Keywords:** integrated pest management, aggregation pheromone, antifeedant, attractant, host plant, semiochemical, cropping management

## Abstract

The Colorado potato beetle (CPB) has been a major insect pest to potato farming for over 150 years and various control methods have been established to reduce its impact on potato fields. Crop rotation and pesticide use are currently the most widely used approaches, although alternative methods are being developed. Here we review the role of various volatile and nonvolatile chemicals involved in behavior changes of CPB that may have potential for their control. First, we describe all volatile and nonvolatile chemicals involved in host plant localization and acceptance by CPB beetles, including glycoalcaloids and host plant volatiles used as kairomones. In the second section, we present the chemical signals used by CPB in intraspecific communication, including sex and aggregation pheromones. Some of these chemicals are used by natural enemies of CPBs to locate their prey and are presented in the third section. The last section of this review is devoted a discussion of the potential of some natural chemicals in biological control of CPB and to approaches that already reached efficient field applications.

## 1. Introduction

The Colorado potato beetle (CPB) *Leptinotarsa decemlineata* (Say), a coleopteran native to Mexico, was first described by Thomas Say in 1824. CPB then dispersed throughout North America in Solanaceae fields [1]. In 1874, the first serious damage in potato fields was reported by Riley [2]. The beetle was reported in Europe for the first time in 1922, probably arriving via cargo ships during World War I and subsequently colonizing all of Europe except for the British Isles and Scandinavia [3,4]. Then, CPB continued to expand eastward and has spread now into central Asia and western China [4].

The CPB is an important defoliating pest of potatoes, *Solanum tuberosum* L., but can also be found on tomatoes or eggplants [5,6]. In late spring, adults emerge from the soil and colonize potato fields in the surroundings. A mated female can lay up to 800 eggs in her lifetime and one, two or even three generations per year can occur depending on climatic conditions [7,8,9]. During their complete larval stage (3 to 4 weeks), CPB larvae consume approximately 40 cm² of potato leaves while adults can ingest up to 10 cm²/day [8].

The most commonly used method of CPB management, the application of insecticides, has resulted in the rapid development of resistance to most of the active substances [10,11,12,13]. Other approaches suggested for control of CPB are listed in Table 1. Among these alternative control approaches, crop rotation, a no cost method, can be effective if there are large distances between the newly planted fields and crops of the previous year [14,15]. Delaying planting time has also been commonly used. Physical barriers have not applied because of the time and energy required to establish them. Mechanical control causes undesirable damage and its efficacy should be improved. Prices of biological control are too high for economic use. The use of transgenic crops is an efficient control method but is currently unacceptable to many consumers [16,17]. Recently, RNA interference was suggested as a new control method but it is still in the experimental stage. Given the limitations of the different control methods and requirements of consumers, chemical ecology may prove to be an alternative method for controlling CPB, through the use of antifeedants or attractive and/or repulsive volatile organic compounds (VOC) deployed to modify chemical interactions of CPB with its environment.

**Table 1:** Different approaches suggested to control the Colorado potato beetle.

*Category*	*Control* *Approaches*	*References*
Cultural modifications	Crop rotation	[14,18]
	Delayed or early planting	[15]
	Trap crops	[15,19]
Physical barriers	Straw mulch ground cover	[20]
	Traps with plastic trenches	[21]
Mechanical control	Use of propane flamers	[22]
	Vacuum collection devices	[23]
Biological control	Predators or parasitoids	[24,25]
	Nematodes	[26,27]
	Fungi	[28]
Genetical modifications	Transgenic plants with *Bacillus thuringiensis*	[29,30]
Molecular biology	RNA interference	[31]

## 2. Chemicals Involved in Host Plant Selection

### 2.1. Role of Glycoalkaloids in Acceptance, Repellency and Defense

The CPB is a specialist herbivore of plants in the family Solanaceae. Hsiao and Fraenkel [32] evaluated the acceptance of 104 plant species and found that only 36 of them were accepted to some degree by CPB. Among the 15 hosts that allow for completion of the CPB life cycle, only four are non-solanaceous plants. Feeding tests with different host plants showed that while potato is generally preferred [32], CPB populations in some regions showed preference for other Solanaceae [33].

**Figure 1:**
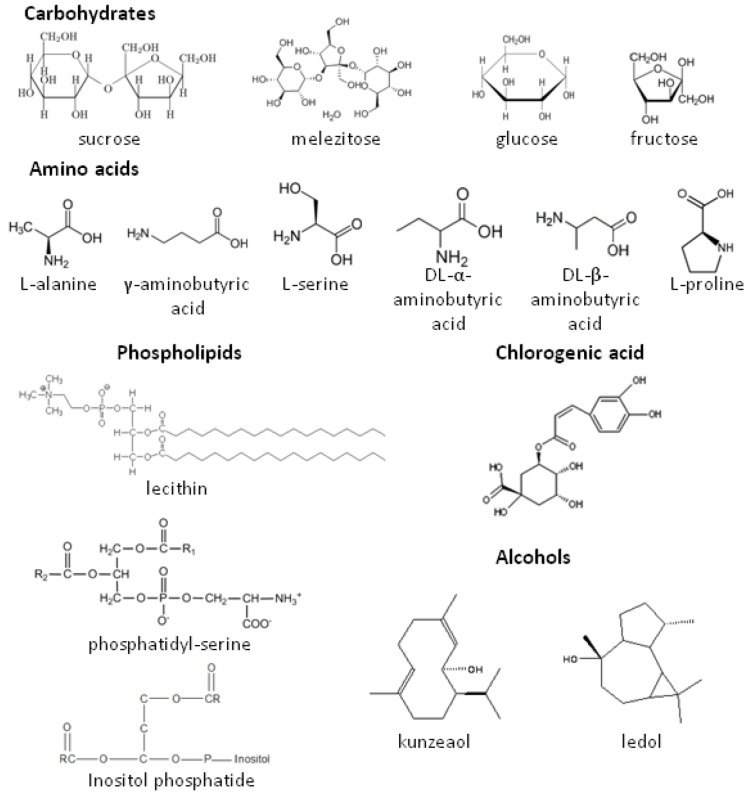
Feeding stimulants of the Colorado potato beetle, *Leptinotarsa decemlineata*.

In phytophagous insects, chemical signals have an important role in host plant recognition and acceptance. For CPB, several feeding stimulants (Figure 1) have been identified by Hsiao and Fraenkel [34] as carbohydrates (sucrose, melezitose, glucose and fructose), amino acids (L-alanine, γ-aminobutyric acid, L-serine, DL-α-aminobutyric acid, DL-β-aminobutyric acid and L-proline), phospholipids (lecithin, phosphatidyl L-serine and inositol phosphatide) and chlorogenic acid [35]. Inorganic salts (KCl, KH_2_PO_4_ and NaCl) may act as co-factors of phagostimulants and enhance feeding [34]. Kunzeaol and ledol, two alcohols present in the leaf surface of potatoes, showed a phagostimulant effect but the concentration applied in bioassays was 5-fold higher than in a natural potato leaf [36].

**Figure 2:**
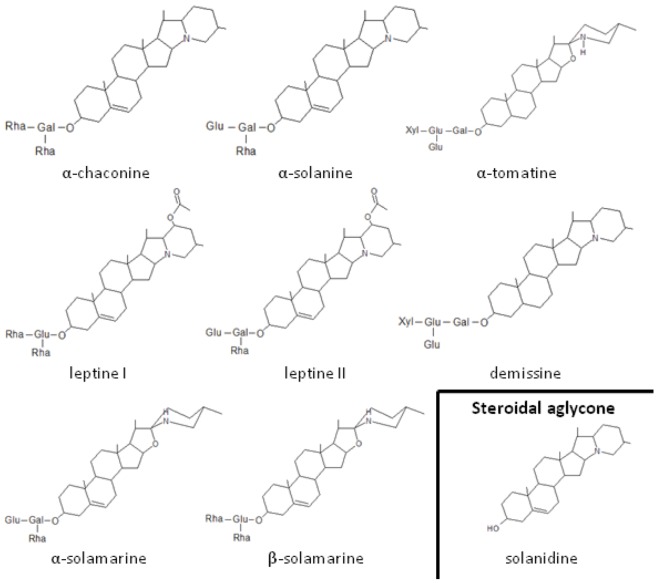
Potential deterrents for Colorado potato beetle, *Leptinotarsa decemlineata*, include glycoalkaloids and a derived aglycone. Abbreviations for sugar group: Rha: Rhamnose; Gal: Galactose; Glu: Glucose; Xyl: Xylose.

Potatoes and other solanaceous plants contain glycoalkaloids [37,38] (Figure 2) that are thought to provide resistance against herbivorous insects including CPB [39]. Solanine, chaconine, tomatine [40], leptines [41] and demissines [42] have been identified as deterrents. Hybridization was used to create new potato progenies containing leptines with increased resistance to beetles [43]. Hollister *et al.* [44] demonstrated a specific dose dependent response to leptine I from a neuron associated with chemosensory hairs on the galea of CPB. Solanine and tomatine did not induce dose dependent responses but modified responses to leptine I and elicited irregular bursts of neural activity. These results are consistent with those of others and provide neural mechanisms for feeding deterrence attributed to these alkaloids [44,45,46].

Various studies have examined the impact of glycoalkaloids on the development of CPB with equivocal results. Hare [47] tested the addition of glycoalkaloids (α and β-solamarine, α-tomatine, α-chaconine and α-solanine) to diets and observed weight gain of larvae after 24 hours. He concluded that larval development was negatively affected by each alkaloid. Kowalski *et al.* [48] studied the effects of five alkaloids (α-tomatine, α-chaconine, α-solanine, leptine I and the steroidal aglycone solanidine) on the development of CPB larvae, from hatching to prepupal stage. They highlighted the adverse effects of leptine I and steroidal aglycone solanidine on the development of the larva (weight gain and time to molt). On the other hand, α-chaconine (at high concentration) and a mix of α-chaconine with α-solanine (at concentrations commonly found in *S. tuberosum*) did not affect the larval growth. In 2007, Lyytinen *et al.* studied three varieties of potatoes in terms of nitrogen content (positive effect on beetle performance expected) and glycoalkaloid concentration (negative effect expected) [49]. Based on larval growth and survival, no significant difference was found in beetle performance related to the glycoalkaloid content of the potato variety. Moreover, Armer [50] showed that fourth instar CPB larvae and adults neither sequester nor metabolize glycoalkaloids (solanine and chaconine). In conclusion, the higher the concentration in glycoalkaloids, the more deterrent the host plant. However, the latter studies indicate that glycoalkaloids do not affect survival of larvae and adults.

Other chemical defense mechanisms against herbivores in plants have also been reported. For example, Kruzmane *et al.* [51] showed that larval regurgitant increased production of ethylene and activity of two plant enzymes, peroxidase and polyphenol oxidase. The larval regurgitant can override proteinase inhibitors produced by the plant to limit the growth of herbivores. Indeed, two studies showed that the regurgitant of larvae prevented these genes transcribing some proteinase inhibitors [52,53]. Another study on gene expression in tomato, *Lycopersicon esculentum* L., showed that CPB larval regurgitant, induced a lower expression of defense genes compared with that induced by larval regurgitant of *Manduca sexta* (L.) (Lepidoptera: Sphingidae) [54].

### 2.2. Role of Plant Volatiles in CPB Orientation

When CPB complete diapause and emerge from soil in spring, they quickly need to find host plants, using their olfactory system to locate a food source. The first observation on the attractive effect of volatile organic compounds (VOC) from potato was made by McIndoo [55] using an olfactometer test. In 1950, Chin also found that larvae were attracted by potato VOCs but at very short distances (<5mm) in an assay without airflow [56]. In a wind tunnel, De Wilde *et al.* [57] showed that CPB adults were: (1) attracted to odors released by potential host-plants (potato, tomato, bittersweet, black nightshade and celery), (2) indifferent to alder, and (3) repelled by grass and dandelion. The attraction by potatoes disappeared with excision of the fourth antennal segment [57]. In another study, Visser and Nielsen [58] showed that CPB were attracted to solanaceous plants and supposed that after contact with the host plant, other mechanisms were involved in final acceptance. Starved males and females were attracted to undamaged potato plants in a wind tunnel [59]. Attraction of CPB toward potato plants was stronger with plants aged from four to eight weeks than with young potato plants [59]. Further, Thiery and Visser [60] showed that hunger was also important. In experiments using a servosphere, fed females exhibited less direct paths to potato VOCs, a decrease of the average speed, and more stops compared to starved females which showed a more distinct attraction to potato VOCs.

**Figure 3:**
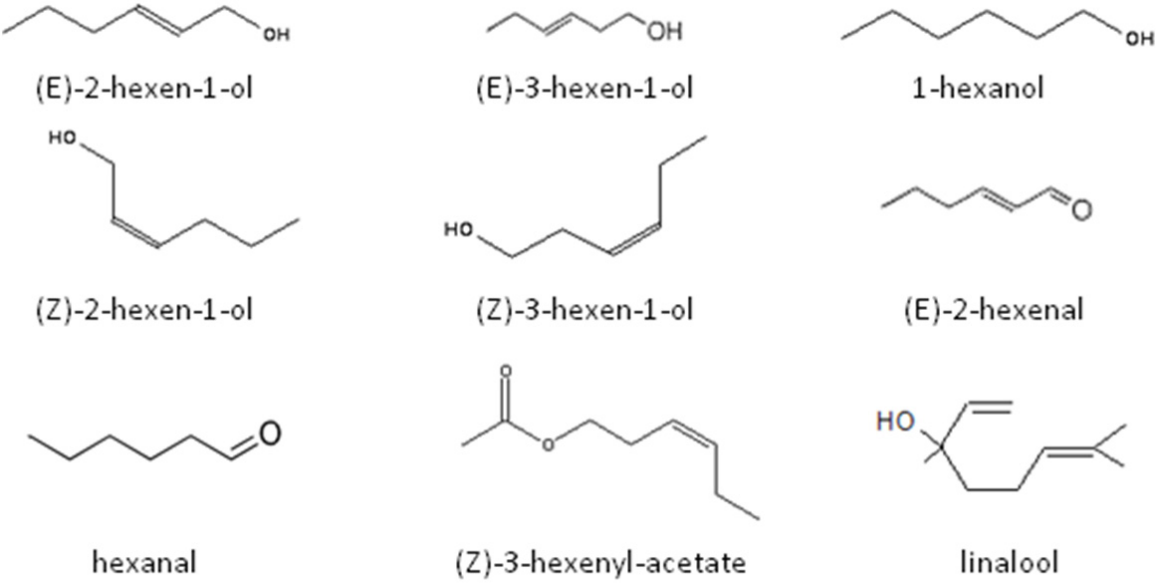
Green leaf volatiles and their isomers from potato as potential chemical signals for the Colorado potato beetle, *Leptinotarsa decemlineata*.

In 1979, Visser *et al.* characterized several VOCs called green leaf volatiles (GLVs) from potato with a successive vacuum steam distillation, freeze concentration, and extraction. (*E*)-2-hexen-l-ol, 1-hexanol, (*Z*)-3-hexen-l-ol, (*E*)-2-hexenal, and linalool were identified as main components of potato odor [61]. Visser [62] used electroantennograms (EAGs) to show that olfactory receptors of CPB adults responded to VOCs including (*E*)-2-hexen-1-ol, (*Z*)-3-hexen-1-ol, 1-hexanol, (*E*)-2-hexenal, hexanal and (*Z*)-3-hexenyl-acetate, *i.e.* GLVs, and to their isomers such as (*E*)-3-hexen-l-ol and (*Z*)-2-hexen-l-ol (Figure 3). Dickens [63] confirmed the EAG results of Visser [62] and demonstrated the importance of sexual maturity for the recognition and attraction of the plant VOCs. The sensitivity of CPB antennae to potato VOCs increased with the number of post emergence days and sexual maturation. Maximal antennal sensitivity was recorded at 6–8 days males and 12–14 days females [63]. VOCs were classified into five groups based on the development and magnitude of the EAGs they elicited: (1) chemicals with a strong response and a weak variability during sexual maturation such as (*Z*)-3-hexen-1-ol and (*E*)-2-hexen-1-ol; (2) chemicals with an intermediate response and slightly increasing response with maturity such as methyl salicylate, nonanal, and (*Z*)-3-hexenyl butyrate; (3) chemicals with a low response and a little variation with maturation, including indole, (±)-linalool, and decanal; (4) chemicals with a weak response and slightly increasing reaction during the maturation such as β-caryophyllene and β-selinene; (5) chemicals with a weak response and a decreasing activity with maturation, including 1,8-cineole, (*R*)-(+)-limonene, (*S*)-(−)-limonene, myrcene, (1*R*)-(+)-a-pinene, (1*S*)-(−)-a-pinene, a-humulene, and (+)-longifolene. Mitchell and McCashin [64] showed that CPB may also taste GLVs. They found that nerve cells housed within the galeal sensilla of both adult and larval CPB responded to primary alcohols (hexanol and heptanol) and other components found among the GLV mixture such as the monounsaturated (*Z*)- and (*E*)-isomers of hexen-1-ol and the six-carbon aldehyde analog, (*E*)-2-hexenal.

GLVs are found in the headspace of most plant species and the fact that beetles distinguish potatoes from other plants is thought to be due to their ability to perceive the proper ratio of GLVs corresponding to potatoes [65]. Indeed, GLVs identified as originating from potato were tested individually and CPB females showed no attractive effect [61]. When potato GLVs were added, one by one, to the original odor cocktail from potato, no attraction was found, demonstrating that the whole cocktail of GLV in a very specific ratio, attract CPB [65]. Using an open air Y-olfactometer, CPB discriminated between two plants on the basis of their VOCs [66]. In this study, CPB preferentially chose potato *versus* tomato and eggplant, and preferred volatiles of the eggplant over the tomato. In field studies, when potatoes were intercropped or surrounded by other plants, the positive anemotaxis disappears because CPB do not perceive the potato odor blend alone. This change in the ratio of individual volatiles within the odorous blend disrupts orientation of CPB [67,68,69], a point that will be discussed further in a context of integrated pest management (IPM).

Wounded potato plants have different emissions of VOCs compared to healthy plants, and changes depend on the nature of the damage: mechanical damage, molecules eliciting defense mechanisms, or CPB-infested plants. Whatever the origin of damage, CPB adults are attracted [70,71,72]. Bolter *et al.* [70] confirmed the results of Visser [59] that old plants were attractive to CPB females while young plants did not attract beetles. When plants were wounded using carborundum powder, VOCs were released that induced positive anemotaxis [70]. Positive anemotaxis was observed when CPB larvae actively fed on foliage, but this behavior disappeared when CPB wounded potatoes were tested 50 min after a feeding period of 30 min. When the feeding duration was longer, attraction was observed for a longer period of time. This study suggested that there are different volatiles inducing attraction. First, VOCs released directly from the wound site and later VOCs in response to herbivorous pests [70]. Similar results were found for damage caused by *Spodoptera exigua* (Hübner) (Lepidoptera: Noctuidae) larvae feeding on potato [70]. Potato plants injured by CPB larvae released an amount of VOCs seven to ten-fold higher than quantities released by healthy plants [70]. Anemotactic behavior of CPB was evaluated for VOCs released by plants that were: (1) damaged by insect feeding (CPB and cabbage looper, *Trichoplusia ni* (Hübner) (Lepidoptera: Noctuidae)), (2) chemically treated with either volicitin, a molecule isolated and identified from beet armyworm larval regurgitant that induces corn seedlings to emit volatiles [73] or methyl jasmonate, a compound that induces synthesis of proteinase inhibitors in plant leaves [74], (3) mechanically damaged [71,72]. Damage by CPB and cabbage looper larval feeding was mimicked by making an incision on the leaf and applying larval regurgitant to the wound. During the study by Schutz *et al.* [72], preference tests showed a greater attraction for: plants damaged and treated with CPB larval regurgitant > mechanically damaged plants > undamaged plants. Another study showed similar results and noted that plants damaged and treated with larval regurgitant (CPB and cabbage looper) or chemicals (volicitin or methyl jasmonate) were attractive for CPB females while undamaged plants were not attractive [71]. Moreover, Schutz Schutz *et al.* [72] demonstrated attraction by β-caryophyllene over a short distance while 2-phenylethanol was attractive at a long distance.

CPB larvae (2nd and 4th instar) are also attracted by the odors from healthy and injured potato plants, without showing any preference for one or the other [75]. A three component blend comprising (±)-linalool, methyl salicylate and (*Z*)-3-hexenyl acetate attracted both larval and adult CPB, the latter being attracted to lower concentrations of this chemical blend [75]. Hammock *et al.* [76] found similar results for CPB larval perception of potato VOCs and noted that larvae reduce their speed in the presence of VOCs, perhaps indicative of the proximity of food resources.

For additional descriptions of setups used in behavioral assays, we recommend the chapter of Hare [77] in "*Methods in Chemical Ecology, Vol. 2*".

## 3. Intraspecific Communication in CPB

### 3.1. Sexual Recognition and Sex Pheromone

In 1969, De Wilde *et al.* hypothesized a sex pheromone in CPB mating behavior [57]. Males were attracted by excised elytra of females; this attraction disappeared when elytra were extracted for 2 h with pentane in a Soxhlet apparatus [78]. This pheromone is likely to act after contact between partners. Jermy and Butt [79] demonstrated that a glass rod, covered by an extract of female elytra, stimulated males to copulate with the rod.

In a greenhouse assay, males moved from a potato plant toward another potato plant 50 cm distant downwind that was infested by females [80]. While this study suggested that a female sex pheromone attracted males over a short distance, one must be cautious when speaking about “sex pheromone” because a chemical has never been identified and the percentage of males attracted by females was relatively low [80].

Dubis *et al.* [81] identified cuticular hydrocarbons of CPB and found the same hydrocarbons in both sexes but with quantitative differences. However, long-chained hydrocarbons were present in higher proportion in females than in males. Physical contact between two individuals of opposite sex also influences foraging behavior toward host plants. After a single contact with another beetle of the opposite sex, CPB remained attracted to potato VOCs for 24 hours, but during the second day, this attraction disappeared. Attraction to VOCs emitted by potato reappeared only after 72 hours. This should be considered as an adaptation to lower attraction for host plant while enhancing reproduction success as suggested by Dickens who observed that during two days, beetles moved less and increases their chances to meet a sexual partner [82]. These studies demonstrated the importance of contact between partners but did not demonstrate the existence of long-range sex pheromone. These studies led some researchers to reconsider the existence of a volatile sex pheromone and to explore other possibilities of long-range pheromones.

### 3.2. Aggregation Pheromone and Aggregation Behavior

**Figure 4:**
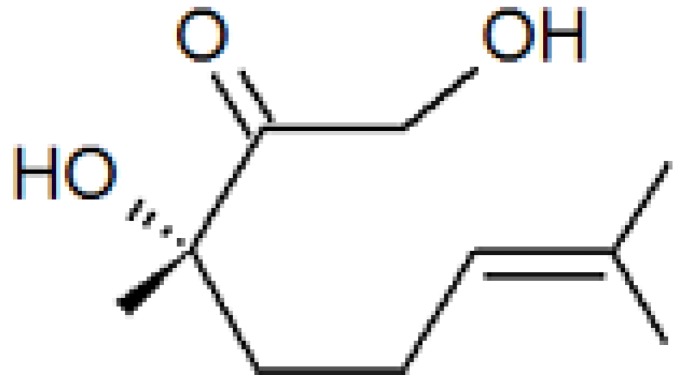
Male-produced aggregation pheromone for Colorado potato beetle, *Leptinotarsa decemlineata*: (S)-3,7-dimethyl-2-oxo-oct-6-ene-1,3-diol or (S)-CPB I.

While several studies were directed to the characterization of a potential female-produced sex pheromone (see above), Dickens *et al.* [83] announced the discovery of an aggregation pheromone produced by males. Using GC-EAD (gas chromatography coupled with an electroantennographic detector) for analysis of aeration extracts of CPB males, active components were identified which elicited responses from olfactory receptors of both males and females. Among these chemicals, a single molecule was only found in volatiles of plants being fed on by male beetles. As only small quantities of the unknown compound were released, it was reasoned that males must regulate release through a feedback loop involving olfactory receptors on the antennae. When the antennae were excised, quantities of the unknown compound increased 40-fold. Topical application of juvenile hormone III (JH III) also stimulated pheromone release to a lesser extent. However, the combination of antennectomy and JH III treatment allowed a 200-fold increase, thus facilitating identification of the compound as (*S*)-3,7-dimethyl-2-oxo-oct-6-ene-1,3-diol, named (*S*)-CPB I (Figure 4) [83]. The biological effect of (*S*)-CPB I was first evaluated in a Y-olfactometer on male and female CPB, both of which were highly attracted. The (*R*) enantiomer and the racemic mixture were not attractive [83]. CPB larvae also seem capable of perceiving the aggregation pheromone produced by adults, but further studies are needed to characterize larval behavior [76]. This aggregation pheromone should be useful in IPM programs as a key component of a potential attracticide (see later).

## 4. Interspecific Interactions

### 4.1. Defensive Chemicals

CPB hemolymph contains a protein called leptinotarsin which is highly toxic when injected directly in insects or vertebrates. However leptinotarsin was found to be less toxic when ingested [84]. In mammals, this protein instantaneously blocks heart muscle cells [84,85,86]. Leptinotarsin also limits reproduction of the pathogenic nematode, *Heterorhabditis marelatus* Liu and Berry, in CPB [87].

When disturbed, adult CPB eject a secretion through defensive glands located on the pronotum and the elytra. The major chemical component of the secretion has been identified as γ-L-glutamyl-L-2-amino-3(Z), 5-hexadienoic acid [88]. Preliminary experiments suggested that this molecule is toxic for *Myrmica rubra* (L.) (Hymenoptera: Formicidae) and deterrent for chickens [88,89].

### 4.2. Volatile Perception by Predators

Dickens [90] analyzed GC-EAD responses of a generalist and a specialist CPB predator, respectively, *Podisus maculiventris* (Say) (Hemiptera: Pentatomidae) and *Perillus bioculatus* (Fabr.) (Hemiptera: Pentatomidae), to constitutive odors and systemic volatiles elicited by CPB feeding. GC-EAD tests showed that, unlike CPB that detect easily GLVs, predators were more sensitive to volatile molecules induced by the systemic reaction of the plant injured by CPB larvae. Other studies on *P. bioculatus* showed the attractant effect of plant volatiles from a CPB infested plant [91,92,93]. CPB damage of plants caused a systemic reaction inducing production of VOCs that attracted third and fifth instars of *P. maculiventris* [94].

### 4.3. Interactions between Different Pest Species

The fact that a potato field is already attacked by another herbivore than CPB may influence the distribution of CPB. Bolter *et al.* [70] first demonstrated attraction of CPB adults to potatoes damaged by heterospecific herbivores, e.g., *S. exigua* larvae. Field cage tests with high densities of leafhoppers affected CPB as: (1) fewer egg masses on the potato foliage were observed, (2) larval development was longer with fewer surviving and (3) freshly emerged adults weighed less [95]. While this competition between the herbivores could be used by farmers to limit colonization by CPB, one must ensure that leafhopper damage does not involve an important yield loss. Furthermore, the idea of placing other pests competitive with CPB on potatoes remains unrealistic as farmers would be unlikely to accept such an approach.

Gosset *et al.* [96] demonstrated that volatiles emitted by injured potato depended on whether the herbivores were piercing-sucking or chewing type feeders. Despite apparently lesser injuries inflicted by the aphid, *Myzus persicae* (Sulzer) (Homoptera: Aphididae), a greater number of volatiles were released, perhaps due to pathogen infection. Aphids were also unaffected by preliminary CPB wounding, suggesting that the presence of CPB does not influence the colonization behavior of *Macrosiphum euphorbiae* (Thomas) (Homoptera: Aphididae) [97].

## 5. Semiochemical-Based IPM Strategies

Recent discoveries have enhanced knowledge of chemical communication in CPB and highlight the potential of semiochemicals as a component of future integrated management strategies. However, the chemical ecology of CPB is not yet completely understood and this incomplete knowledge makes semiochemical-based approaches inefficient when compared to traditional insecticide treatments. However, alternative strategies have potential in the control of CPB populations and include: (1) disorientation of CPB adults by masking potato VOCs with intercropping cultures; (2) use of synthetic mixtures of volatiles and/or aggregation pheromone to trap beetles; (3) antifeedant sprays on potatoes; (4) increase, with genetic manipulations, the natural capacity of the plant to recognize the presence of CPB through chemical signals, thus triggering defense mechanisms. These tools are summarized in Table 2.

### 5.1. Disorientation of CPB Adults with Masking Odors

Intercropping represents an efficient method to repel and/or confuse CPB foraging for host plants. Among the 13 identified compounds from tansy oil, five were repellents (α- and γ-terpinene, α,β-thujone, dihydrocarvone and carvone) and one compound (α-pinene) was an attractant [98]. When tansy was used as an intercrop in potato fields, a 60%–100% decrease in the number of beetles present in the fields was observed [99]. Two other studies have shown that potato VOCs mixed with VOCs from tomato or cabbage, disrupted the searching behavior of CPB females for host plants [68,69].

These initial results demonstrate the exciting potential of intercropping and support preliminary laboratory studies by Visser and Ave [65] that suggested that host orientation could be disrupted by modification of the ratio VOCs. Moreover, few intercropping models were studied and further investigations should be considered with other plant species.

**Table 2:** Different approaches suggested to control the Colorado potato beetle.

*Name*	*Schemas*	*Brief description*
*Masking odors*	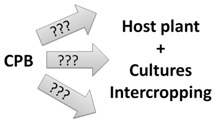	*CPB cannot detect its host plant because GLVs of potatoes were mixing with others odors from cultures intercropping*
*Trap crops*	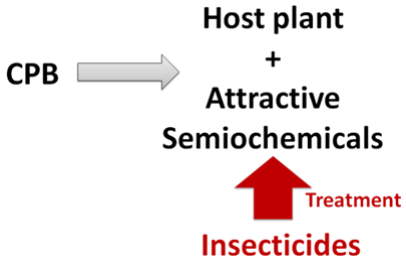	*CPB are attracted in a specific part of the field with semiochemicals and only this part is treated to eliminate the beetles*
*Antifeedants*	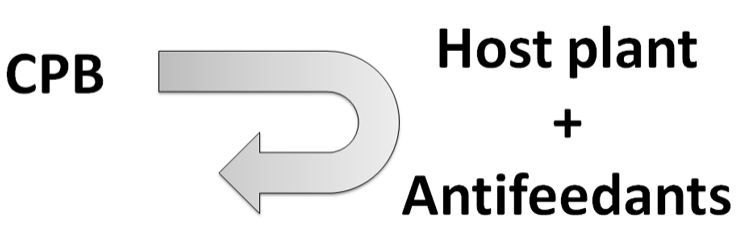	*Chemical molecules were sprayed on potatoes to deter CPB feeding and damages are lesser on potato foliage*
*Genetic manipulations*	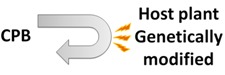	*Potatoes are genetically modified to detect CPB presence and to express more their defense mechanisms to prevent CPB colonization*

### 5.2. Use of Attractants and Aggregation Pheromone

The use of semiochemical attractants to improve insecticide treatments should be considered as an innovative approach of CPB management. Chewing insects are indeed more sensitive to VOCs released by their host plants because the damage they induce in plant tissues increases the release of these compounds [100]. The difficulty consists in finding a cocktail of natural odors within which the quantitative proportion of each compound is as close as possible to that of the naturally emitted blend [65]. The challenge therefore consists in finding the appropriate molecules and their ratio, instead of trying to include as many compounds in the mixture as possible [100].

The first synthetic blend to show potential in CPB management was comprised of (*E*)-2-hexen-1-ol, (*Z*)-3-hexen-1-ol, nonanal, (±)-linalool and methyl salicylate prepared in paraffin oil [90]. This blend, placed in competition with pure air attracted CPB adults in behavioral assays with a “T” choice test in the laboratory. For better efficiency, the proportion of (*E*)-2-hexen-1-ol and (*Z*)-3-hexen-1-ol were 10-fold lower compared to the three other compounds [90]. Dickens [75] also reported a synthetic blend of three compounds, (*Z*)-3-hexenyl acetate, (±)-linalool and methyl salicylate, which attracted second and fourth larval instars. This blend attracted CPB adults with a 10-fold lower concentration for females and 100-fold lower for males. Yet, an earlier study of [101] could not show attraction of females with this last blend. This attractive blend for all stages of CPB has a very interesting potential for creating attracticides in the field [75].

The synthetic blend made of (*Z*)-3-hexenyl acetate, (±)-linalool and methyl salicylate, firstly reported in Dickens study [75] was evaluated in a greenhouse where it attracted newly emerged and 5-day-old adults when released at a rate of 57 μg/h [102]. However, habituation was observed in the beetles that were exposed more than 12 hours to this mixture. Initial field tests confirmed the effect of the attractant blend and its usefulness for semiochemical-based control [103]. Pitfall traps baited with the attractant captured a greater number of CPB in potato fields. Moreover, fewer egg masses and larvae were observed in neighboring untreated crops. The use of trap crops with the attractive blend allowed a decrease of 44% insecticides for similar yields to the conventional method [103].

The first field experiments with the aggregation pheromone, (*S*)-CPB I, were performed by Kuhar *et al.* [104]. They placed pitfalls traps baited with the aggregation pheromone in a 0.5-ha land section cultivated for a good soil uniformity and wherein they released 400 CPB adults in the centre. More than 5-fold numbers of CPB adults were captured in traps baited with septa releasing the pheromone than in control traps. This significant difference in treatment numbers disappeared after 5 days presumably due to evaporation loss or degradation of the pheromone. In the second part of their study, they showed that fewer CPB eggs and larvae were present in plots bordered with pheromone-treated plots than in field plots bordered with untreated rows [104]. While these preliminary results offer promise for use of the pheromone in the field, more research is needed before practical applications may be available.

In laboratory tests with an vertical Y-olfactometer, CPB adults were confronted with a cocktail of three volatiles, (*Z*)-3-hexenyl acetate, (±)-linalool and methyl salicylate, opposite to the aggregation pheromone [105]. Half of the tested CPB adults chose the aggregation pheromone side of the olfactometer and the other half chose the side connected to the plant volatiles. This indicates that CPB adults were unable to choose between plant VOCs blend and their aggregation pheromone in this vertical bioassay. However, in mowed field assays, the aggregation pheromone coupled with the blend of three odors attracted significantly more CPB adults in comparison with the plant odors blend alone [105].

Another study in fields was made with the synthetic blend of three plant odors [(*Z*)-3-hexenyl acetate, (±)-linalool and methyl salicylate] [106]. The synthetic blend was coupled with a pyrethroid insecticide at different concentrations. A decrease in first and second instar CPB was observed with an application of 2% or 6% active ingredient permethrin coupled with the odorous blend but this method did not significantly decrease older larval stages because the insecticides were more efficacious on young larval stages [107]. However, the use of this attracticide showed the same control efficiency as the commercial insecticide while using 92% less insecticide by active ingredient [106]. As many CPB populations in the U.S. have developed resistance to permethrin [108], the authors suggested imidacloprid as a replacement [106].

When considering use of chemical signals for manipulation of the behavior of CPB or other insects, it is important to understand that behavior of insects is a result of integration of multimodal signals in the environment of which olfaction represents only one of the input channels. For example, CPB walking on a servosphere orient to the aggregation pheromone in darkness [109]. However, in the presence of yellow light, a known attractant for CPB, orientation to the pheromone is abolished [110]. Thus, better understanding the complexities of the behavior of CPB and other insects to multimodal signals will enhance design and deployment of management strategies employing naturally-occurring chemical signals.

The main difficulty with semiochemicals is ensuring a controlled release during a long period. This depends of the active molecule and its release rate kinetic. The knowledge of these molecules is thus essential. Other parameters such as UV light, oxygen, pluviometry and temperature could also alter semiochemicals. Therefore, the choice of the dispenser is also important to control the release and to prevent molecule degradation [111].

### 5.3. Antifeedants

Antifeedants to deter CPB adults and larvae include a wide variety of chemicals. The first chemical deterrents were identified by Hsiao and Fraenkel [112]. Other studies identified a multitude of additional antifeedants including: (1) constituents of some plants from the sagebrush community [113], (2) hydroxides which also act as fungicides [114], alcohol extracts of the leaves and bark of *Quercus alba* L. [115], (3) limonin [116], (4) α-mangostin [117], (5) sesquiterpenes [118], (6) terpenoid lactones [119], and (7) extracts from various plants [120,121] including wild species of potatoes [122,123]. Such molecules and blends of chemicals not only reduce feeding but also may deter oviposition by females as shown for citrus limonoids [124,125]. A phagorepellent effect was also found for leaf surface molecules specific to commercial varieties of *S. tuberosum*. However, the deterrent effect was observed when concentration of these molecules was 10-fold higher than in natural leaves [126]. One should be cautious when discussing antifeedants as physical properties of the plant (e.g., trichomes) and systemic modification of the plant due to the application of an external molecule may also play a deterrent role, an indirect antifeedant effect [127].

Other field assays were conducted in Hungary by Szentesi [128] in which crops were treated with a 2% Bordeaux mixture during two consecutive weeks. This treatment resulted in significantly more eggs laid on the treated potatoes. The unequal distribution of eggs may have been caused by: (1) a decrease of foliage surface, (2) the presence of different larval instars or (3) the presence of larval and adult feces on unsprayed potatoes, thus highlighting the potential of field areas treated as egg traps [128]. In another study, fields treated regularly with fungicides, triphenyltin hydroxyde (TPTH) or copper hydroxyde (Cu[OH]_2_), had fewer CPB larvae, consistent with these fungicides acting as deterrents. The authors suggested a regular application of TPTH to reduce the use of insecticides and decrease cost [129].

A neem-seed extract showed good efficiency to control CPB in fields but the magnitude of the effects depended on the dominant CPB life stage present when the application was made [130]. Yields were in some cases commercially acceptable, thus suggesting that neem seed extract applied in the potato growing season may control young larvae without insecticides. In 2006, Moreau *et al.* [131] showed that application of 2% neem reduced CPB densities and defoliation resulting in increased tuber yields. Azadirachtin is the main active molecule from neem extract and a field evaluation with this natural antifeedant showed satisfactory results for reduction of plant damages [132].

Fields tests on the deterrent effect of citrus limonoids were made during three seasons in Maine. Colonizing CPB adults were fewer in limonoid treated plots, with a decrease in egg masses and smaller numbers of larvae [133]. These results were opposite with those of Szentesi [128] who showed that oviposition increased on potato plants treated with an antifeedant, 2% Bordeaux mixture. Murray *et al.* [134] showed that two applications of a high concentration (10.8 kg/ha) and three applications at a lower dose (3,6 kg/ha) of citrus limonoids were sufficient to maintain the rate of defoliation below 60%, allowing acceptable yields. This study seemed to confirm the potential of limonoids for the management of young larvae at the beginning of the potato growing season [125].

Despite decades of study, the commercialization of antifeedants has been generally unsuccessful because: (1) they were considered as synthetic insecticides, (2) the life span of an antifeedant may vary depending on weather conditions, (3) they may take a long time before acting and (4) they do not always kill or deter target insects [133].

### 5.4. Genetic Manipulations

Recently there has been interest in genetic manipulation of potato to enhance expression of deterrent compounds. Hybrid assays were conducted with *S. tuberosum* and wild potato species to incorporate the gene expressing leptine and additional glycoalkaloids, to create commercial varieties having enhanced tuber yields and repellent activity [135,136]. Another possibility of genetically modified (GM) potatoes would take advantage of their hypersensitivity to chemical molecules associated with the presence of CPB eggs. In 1972, Wegorek & Dubniak [137] observed necrotic areas near oviposition sites on potato leaves in Polish fields. Since no microorganisms were detected near the plant necrosis, the authors supposed that the necrosis was due to female substances secreted during oviposition. Balbyshev & Lorenzen [138] observed a hybrid potato species with a hypersensitive response to egg deposition; a necrosis around eggs followed by the detachment of this part of the leaf. As neither bacteria nor microorganisms were found, they supposed a chemical associated with eggs was responsible for the defoliation. The consequence of this resistance mechanism was that egg masses fell on the soil, thus increasing predation by soil-dwelling predators such as carabids [138].

## 6. Conclusions

Today, research is still being conducted to better understand CPB biology in an effort to devise new control techniques. Toxic chemical treatments have been the best method to control CPB despite continual development of resistance. Now because of the inevitable decline of effective insecticide treatments, research should focus even more on the development of new control methods and approaches. Some methods such as cultivating GM plants are not seen positively by consumers, and farmers have abandoned them due to a lack of buyers [139]. Among the various alternative control techniques, chemical signals seem to be promising, especially when coupled with the use of natural predators and targeted pesticide treatments. Thus, it is necessary to continue to explore alternative control methods using semiochemicals and important to better understand behaviors generated by these semiochemicals.

The genetic variability between CPB populations, which seems to be greater for U.S. populations than for those in Europe [140], is also important to consider. Effectively, CPB have shown metabolic adaptations for: (1) insecticide resistance [10,141], (2) range expansion [142], and (3) different Solanaceous hosts [33,143,144]. One study on two different CPB populations showed a difference of intensity in perception of VOCs between Utah and Wageningen populations [145]. Differences in perception of VOCs and perhaps other chemical signals between CPB populations may be important for the development of semiochemical-based control strategies and merit more investigations.
